# The *i*^2^*Snake* Robotic Platform for Endoscopic Surgery

**DOI:** 10.1007/s10439-018-2066-y

**Published:** 2018-06-11

**Authors:** Pierre Berthet-Rayne, Gauthier Gras, Konrad Leibrandt, Piyamate Wisanuvej, Andreas Schmitz, Carlo A. Seneci, Guang-Zhong Yang

**Affiliations:** Department of Computing, The Hamlyn Centre for Robotic Surgery, Imperial College London, South Kensington Campus, London, SW7 2AZ UK

**Keywords:** Medical robotics, Minimally invasive surgery, Endoscopy, Snake-like, Tendon-driven, Teleoperation

## Abstract

Endoscopic procedures have transformed minimally invasive surgery as they allow the examination and intervention on a patient’s anatomy through natural orifices, without the need for external incisions. However, the complexity of anatomical pathways and the limited dexterity of existing instruments, limit such procedures mainly to diagnosis and biopsies. This paper proposes a new robotic platform: the Intuitive imaging sensing navigated and kinematically enhanced ($i^{2}Snake$) robot that aims to improve the field of endoscopic surgery. The proposed robotic platform includes a snake-like robotic endoscope equipped with a camera, a light-source and two robotic instruments, supported with a robotic arm for global positioning and for insertion of the $i^{2}Snake,$ and a master interface for master–slave teleoperation. The proposed robotic platform design focuses on ergonomics and intuitive control. The control workflow was first validated in simulation and then implemented on the robotic platform. The results are consistent with the simulation and show the clear clinical potential of the system. Limitations such as tendon backlash and elongation over time will be further investigated by means of combined hardware and software solutions. In conclusion, the proposed system contributes to the field of endoscopic surgical robots and could allow to perform more complex endoscopic surgical procedures while reducing patient trauma and recovery time.

## Introduction

Endoscopes are generally composed of a flexible proximal passive section, and an active distal tip that is remotely actuated by tendons and actuation wheels located on the handle of the device. They are typically equipped with a camera, a light source, a suction/irrigation channel and an instrument channel mainly used for biopsies. Standard endoscopic procedures have the advantage that they do not require incisions to introduce the endoscope inside the anatomy, hence reducing the patient trauma and discomfort, thus reducing the risk of complications.^17^ Currently, endoscopes are mainly used for diagnosis and biopsy, rather than complex surgical procedures. This is largely due to technical limitations such as low stability of the tip, the need for manual actuation, and reduced dexterity of the instruments. Typical instruments have 3 degrees of freedom (DOF) and consist of insertion, rotation and grasping. Confined workspace and limited visibility further limit the usability of these systems and require a highly skilled endoscopist to perform dexterous tasks. In this context, there is a need for a new generation of endoscopic systems that use robotics to perform more complex procedures such as Natural Orifice Transluminal Endoscopic Surgery: NOTES procedures.^6^

**Figure 1:**
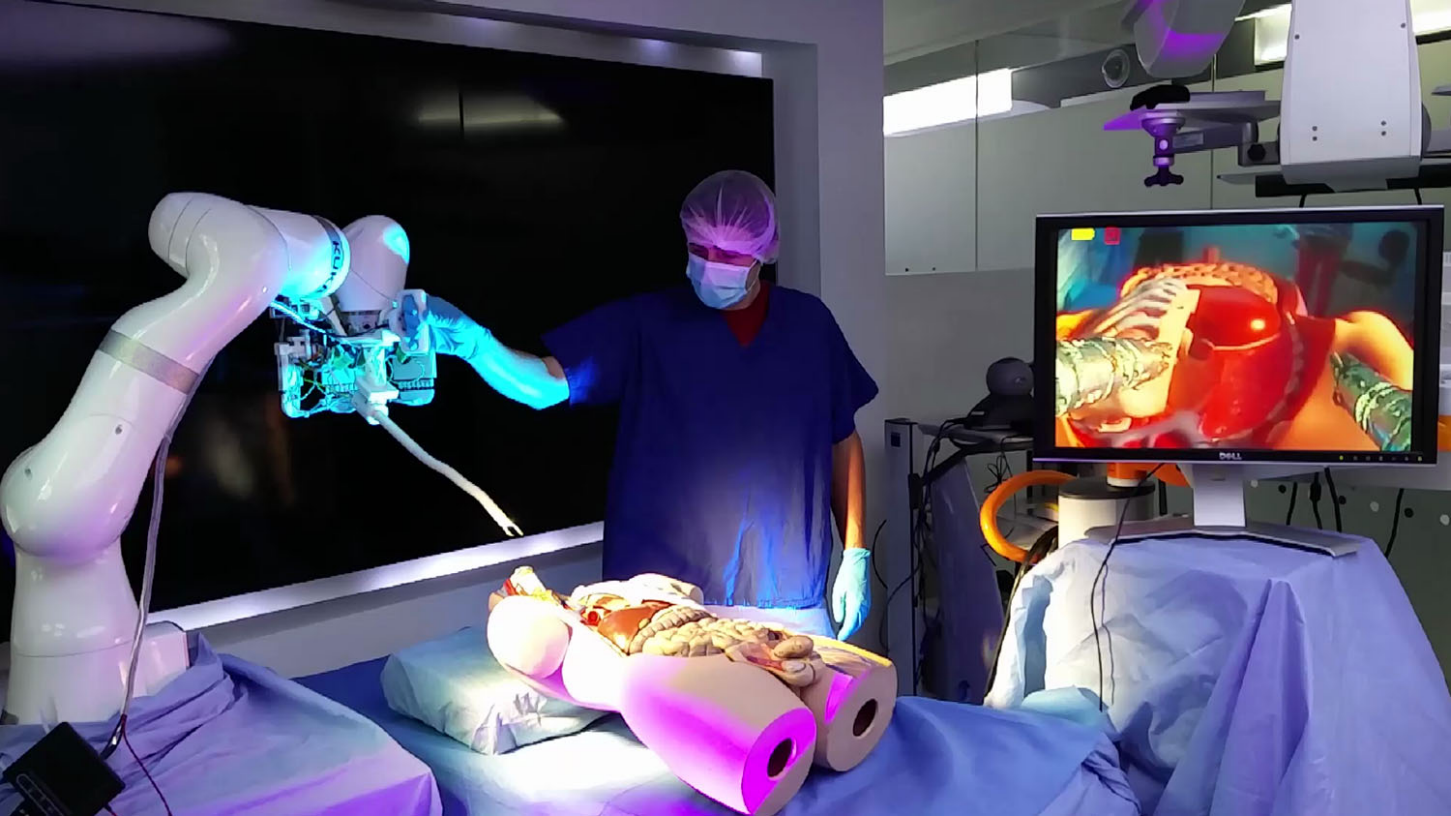
The Intuitive Imaging Sensing Navigated and Kinematically Enhanced Robotic Platform for ENT Surgery: The $i^{2}Snake.$ This platform was developed for ENT surgical applications. The system is composed of a KUKA arm, a snake-like robot equipped with a camera, a light source and two robotic instruments. The monitor on the right displays the live view from the embedded camera.

There are several existing devices that aim to provide a dual arm endoscopic platform.^26^ This introduction will focus on systems that have robotic actuation and at least two instruments, as summarized in Table 1. The first challenge that arises in robotic endoscopes is the question of the outer body actuation under strict size constraints. If the outer body is fully actuated, a large number of DOF are necessary to navigate through natural pathways. In the case of the HARP system,^4^ two concentric articulated snakes are used to perform ‘follow-the-leader’ navigation. This allows the system to follow tortuous pathways, but once the shape is set, it cannot be modified without full retraction of the device. Having complex inner structures also limits the available space for inner instrument channels. The HARP system evolved into the FLEX clinical system (Medrobotics, USA) which uses two manual instruments situated outside the robot, at the cost of an increased overall diameter of the system. The LESS system^11^ uses a phase change material to control a variable stiffness insertion tube that acts as an actuated port. Two instruments are then deployed to perform the surgery, but their actuation is manual. Other approaches investigate a different design by adding two robotic arms to a standard endoscope such as the MASTER system.^13^ This device offers extra dexterity to the surgeon by providing two 5DOF instruments to perform complex tissue manipulation but results in a large outer diameter of 25 mm. Another platform called the IREP^25^ can provide bi-manual robotic tools together with vision and light in a compact 15 mm diameter. This system, however, was designed for single port access surgery and has a rigid body preventing its use for endoscopic applications. The STRASS system^27^ uses a modified Anubiscope (Karl Storz, Germany) controlled robotically. The system provides the surgeon with two 5DOF instruments in a compact 18 mm diameter. The system functions as a typical endoscope with a passive proximal section and an actuated tip. The insertion has to be performed manually, and once the surgical site is reached, the robotic actuation is engaged allowing the control of the instruments and the tip of the endoscope. There exists many other snake-like robots targeting minimally invasive surgical (MIS) applications.^3^,^23^ The work presented in this paper proposes an Intuitive Imaging Sensing Navigated and Kinematically Enhanced Robotic Platform for Ear–Nose–Throat (ENT) Surgery: The $i^{2}Snake,$ combining features such as fully active robotic body, dual robotic instruments, vision and light in a compact and portable design as presented in Fig. 1. The proposed system focuses on intuitive control and ergonomics and was specifically designed for ENT surgical applications such as tumour resection, sleep-apnea surgery and ultimately more complex procedures such as endoscopic submucosal dissection (ESD)^16^ and peroral endoscopic myotomy (POEM).^5^

**Table 1:** System comparison.

System	Body	Carrier	Instruments	Application
HARP	Flexible	Robotic	Manual	Natural orifice
LESS	Flexible	Robotic	Manual	Single port
MASTER	Flexible	Manual	Robotic	Endoscopy
IREP	Rigid	Robotic	Robotic	Single port
STRASS	Flexible	Manual	Robotic	Endoscopy
$i^{2}Snake$	Flexible	Robotic	Robotic	Endoscopy

## Materials and Methods

### System Overview

**Figure 2:**
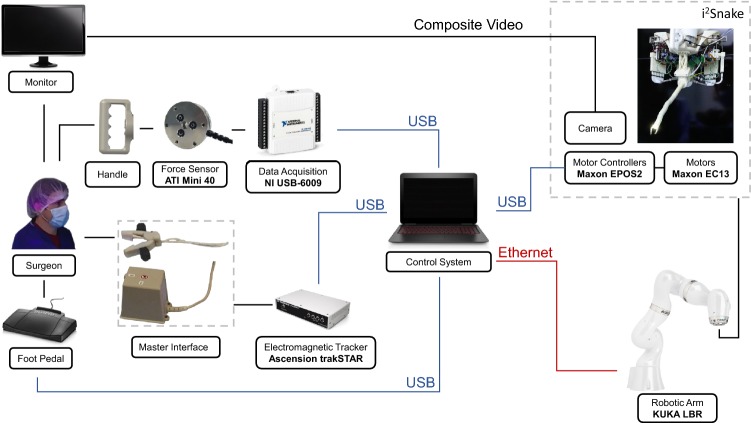
System architecture of the $i^{2}Snake$ robotic platform.

The proposed robotic platform for ENT surgery is composed of five main components. A $i^{2}Snake$ robotic endoscope, an industrial robotic arm to support the $i^{2}Snake,$ two robotic instruments for tissue manipulation, a force sensor with a handle to position the platform and a master interface to teleoperate the system. Each component’s function is described in the following section. The architecture of the robotic platform is shown in Fig. 2.

#### The $i^{2}Snake$ Robot

The $i^{2}Snake$ robotic endoscope is an evolution of the previous *iSnake* system.^20^,^21^ While the *iSnake* concept of a robotic platform for endoscopic surgery has not changed, the $i^{2}Snake$ design has been considerably improved between the two versions. The initial *iSnake* robot was designed for single port surgery. The *iSnake* used a smart combination of embedded micro-motors and local tendon in each joint allowing large joint range and dexterity. The design was compact with narrow internal instrument channels. However, the main drawback was the limited manipulation force available at the tip of the robot, due to the limited torque output of the micro-motors. In this context, a new version, the $i^{2}Snake,$ was developed. the $i^{2}Snake$ currently features a fully tendon-driven control architecture with actuation motors located outside of the body, for both the snake platform and its bimanual instruments. Therefore, the optimal combination of flexibility and force delivery at the tip provides the $i^{2}Snake$ with great potential for ENT surgery. The snake body is composed of 13 stainless steel vertebrae manufactured with a Mlab Cusing 3D printer (Conceptlaser, Germany). The articulations between each vertebra use rolling-joints arranged orthogonally allowing to move in 3D. The outer diameter is 16 mm and the overall length is 36.6 cm. The structure of the body is hollow, allowing room for four 4.0 mm channels. The channels can be used for a camera with an embedded light source, two robotic instruments and the fourth channel can be used for suction/irrigation or a third robotic instrument. The camera used is a 3 mm camera module with a resolution of $640\times 480$ pixels. Although the robot has thirteen vertebrae, 7DOF are used for actuation. The $i^{2}Snake$ kinematics was presented in previous work^2^ but can be summarized as follows: the first DOF starting from the base is a revolute joint rotating all the articulations of the snake robot. The 13 vertebrae following the base joint make up 12 rolling-joints. Pairs of rolling-joints moving in the same plane are coupled mechanically. This results in three actuated sections named proximal, middle and distal, that each has 2DOF. All joints combined, the robot has 7DOF, can fully retro-flex and form S-shapes. Each section is actuated by 8 stainless steel tendons, resulting in 24 tendons for the articulated body plus two tendons for the base rotation. In total, 26 tendons are actuated by 7 brushless EC 13 motors (Maxon Motor, Switzerland). The coupling between the motors and the tendons is performed using capstans in an antagonistic configuration: as a tendon is being pulled, its opposite side is released. The control of the snake robot is performed using inverse kinematics (IK) described in the system control section.

#### Flexible Robotic Instruments

**Figure 3:**
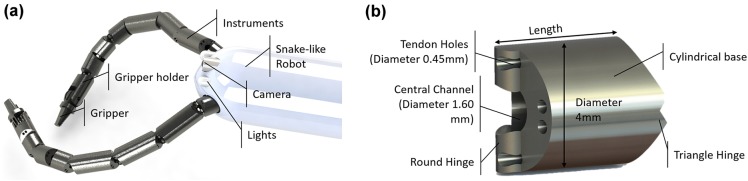
The instruments of the $i^{2}Snake$. (a) Instruments with grippers deployed from the head of the $i^{2}Snake$. (b) One of the links from which the instruments are build.

The internal channels of the $i^{2}Snake$ can accommodate tendon-driven instruments with a diameter of 3.8 mm. The instruments, depicted in Fig. 3a, run through the snake body, are deployed at the tip and can be controlled with the same master interface as the $i^{2}Snake$. The instruments are designed to enhance triangulation, while improving the ergonomics of the system by allowing the surgeon to perceive the instruments as an extension of their arms, and perform the procedure naturally. Various instrument end-effectors can be used, including surgical graspers, scissors, monopolar electrocautery or a ${\text {CO}}_{2}$ laser^18^ fiber for tissue ablation. In the context of this paper, only standard graspers were manufactured. The instruments consist of an alternate series of rigid links and hinges, which are arranged orthogonally to allow for horizontal and vertical motion. The current prototype provides 5DOF with each joint requiring one pair of antagonistically actuated tendons. The instruments use a flexible shaft to adapt to the shape of the $i^{2}Snake$. It guides the tendons that connect the motor unit, (EC 13 motors and EPOS2 controllers, Maxon Motor, Switzerland) located at the proximal end, to the articulated distal tip. The flexible shaft is designed to minimize cross-talk between the snake body movements and the instrument’s tendons. Each link shares the same basic geometry (Fig.  3b). It consists of a cylinder shape with a round and a triangular hinge. The triangular–circular hinge configuration is designed to reduce the friction during motion. Each link provides holes for passing the actuation tendons of the flexible shaft and a central lumen for the tendons of the end-effector, or to pass a laser or imaging fiber.

#### Robotic Arm

The robotic arm is used to hold the $i^{2}Snake$ robotic endoscope.^19^ A LBR iiwa 14 robot (KUKA, Germany) with 7DOF was used. The KUKA robot has three main functions:

- Global positioning of the $i^{2}Snake$ at the mouth entry point,
- Insertion motion along the direction of the snake-like robot,
- Manual collision prevention in a cluttered environment.

In the ‘global positioning mode’, the KUKA robot is controlled by hand in compliant mode, using a handle equipped with a force sensor. This allows the operator to intuitively position the $i^{2}Snake$ robot close to the mouth cavity of the patient. Both the position and orientation are controlled with the handle. The orientation of the snake-like robot is important as it determines the motion direction during the insertion phase.

Once in the ‘teleoperation mode’, the KUKA robot provides an extra prismatic DOF to the $i^{2}Snake,$ allowing it to navigate inside the patient through the mouth cavity. All the 7DOF of the KUKA robot are used to provide the insertion motion with the desired orientation. The motion of the snake robot is combined with the motion of the KUKA using IK control for a more intuitive teleoperation and is solved in two steps described in the system control section.

In the ‘collision prevention mode’, the operator can manually re-adjust the position of the KUKA robot’s joints without affecting the $i^{2}Snake$ position or orientation. This function was designed to provide the surgical team with better access to the patient and to overcome the problem of instrument collision with other equipment in the room such as light, monitors, *etc*. Once in this mode, the operator can move the robot away from other instruments in the operating room by just pushing or pulling manually on the handle. The robot will then move in null-space, in the direction of the force, leaving the snake position and orientation undisturbed.

#### Master Interface

The master interface used to teleoperate the robotic platform consists of a hand-held gripper and a set of three pedals as shown in Fig. 2. The gripper is equipped with two 6DOF electromagnetic (EM) markers tracked using a trakSTAR system (Ascension Technology Corporation, NDI, Canada). These allow the user to intuitively control the robot’s position, orientation and grasping (while controlling the instruments) with a convenient large workspace > 1.5 m and a precision of 1.4 mm. Motion scaling is used to increase the precision of the operator’s motions and can be adjusted in software. The set of three pedals used allows the operator to switch between the three control modes of the robotic platform: global positioning mode, teleoperation mode, and collision prevention mode. Pressing a pedal will select the corresponding mode, and the mode will stay active until the pedal is released. While using the global positioning mode, releasing the pedal will clutch the system, allowing the operator to reposition his hands as in traditional master–slave teleoperated surgical systems.

#### Force Sensor

The hands-on manipulation implemented in ‘global positioning mode’ allows the user to cooperatively move the KUKA robot by hand to a desired location and orientation. To sense the user’s manipulation force, a 6DOF force/torque sensor is installed between the robot arm end effector and the handle. The placement of the sensor is depicted in Fig. 2. The control scheme for hands-on manipulation is implemented by commanding the KUKA robot using the data from the sensor. Due to the specific placement of the sensor, the $i^{2}Snake$’s weight does not affect the force measurement. Therefore, the weight compensation described in “Global Positioning” section renders the mass of the whole system transparent to the user during manipulation, and the tool is able to maintain its position when released. Moreover, this configuration does not require mass parameters, allowing quick tool exchange without mass re-calibration.^24^ To measure the force and torque, a Mini 40 (ATI Industrial Automation, USA) sensor is used. A USB data acquisition device, USB-6009 (NI, USA) is used to convert the analogue measurement to digital format.

### System Control

The control of all the components of the system is performed on a single off-the-shelf computer. Two different types of computers were tested: a Desktop PC and a Laptop PC to improve the portability of the system. The desktop computer was an Elitedesk (HP, USA) with an i7-4790 CPU, 16.0 Gb of RAM and USB 3.0. The laptop computer was an Omen (HP, USA) with an i7-6700HQ CPU, 16.0 Gb of RAM and USB 3.0. Both computers offered similar performances in terms of computing power, although the Ethernet communication to the KUKA robot was slower with the laptop. The control software running on the computer performs all the following tasks, as represented in Fig. 2:

- Read the master interface information (at 100 Hz),
- Read the foot pedal (event-driven),
- Read the force sensor information (at 8 kHz),
- Control the $i^{2}Snake$ motors and instruments (at 100 Hz),
- Compute the IK of the snake system and the virtual joints (> 1 kHz),
- Communicate with the KUKA robot (at 1 kHz),
- Compute IK of the KUKA robot (> 50 kHz).

#### Combined $i^{2}Snake$-KUKA Control

Considering a remote center of motion (RCM) during manipulation is a common requirement of surgical robots. This is due to the incision/insertion point limiting the motion of the instrument being inserted. Many surgical systems are mechanically constrained to the RCM, which provides explicit safety. Other approaches are based on software implementations, which can provide more flexibility in the control. Software-based optimization use additional task-space objectives to minimize the deviation of the robot shaft to the incision point.^1^,^12^ However, they either do not guarantee that the robot shaft extends through the incision point or they consider end-effector pose conformance only as secondary objectives which can lead to increased tracking errors.

**Figure 4:**
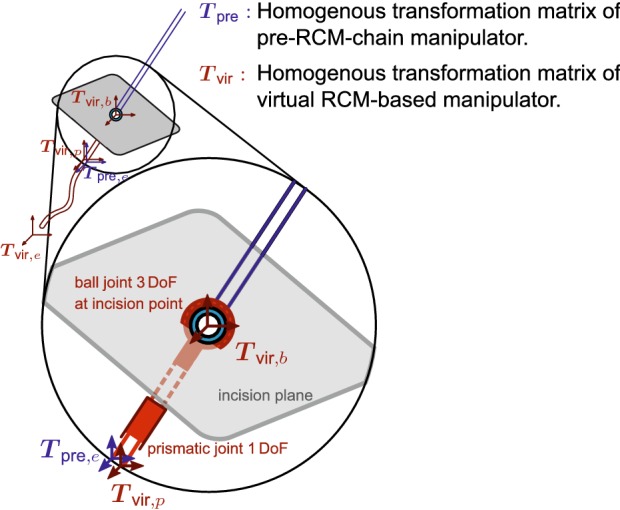
Virtual RCM-based instrument. Separation in two kinematic chains. Red: virtual RCM-based chain, blue: pre-RCM-based chain.

An alternative is presented in the following, where $${\dot{\varvec{x}}}_{{\mathcal {C}},{\mathcal {F}}}$$ represents the 6DOF velocity of frame $${\mathcal {F}}$$ of chain $${\mathcal {C}},$$$${\varvec{T}}_{{\mathcal {C}},{\mathcal {F}}}$$ donates the homogeneous transformation matrix of frame $${\mathcal {F}}$$ of chain $${\mathcal {C}},$$$${\varvec{q}}_{\mathcal {C}}$$ symbolizes the joint-values of chain $${\mathcal {C}},$$ and $${\varvec{J}}_{\mathcal {C}}$$ represents the end-effector Jacobian matrix of chain $${\mathcal {C}}$$. The problem is separated into task-space goals and RCM constraint. This is achieved by using two independently defined kinematic chains. One is the pre-RCM-chain and the other is a virtual RCM-based chain. The virtual RCM-based chain is centered on the RCM and is further divided into a 4DOF base and the post-RCM-chain, as shown in Fig. 4. The 4DOF base consists of a ball joint allowing 3DOF rotations at the RCM followed by a 1DOF prismatic joint. This redefinition and separation of the kinematic chain enables RCM constrained inverse kinematic. Like the Jacobian based approaches, it is easily applicable to any kinematic chain.

Since the real manipulator is a single kinematic chain the problem of solving two independent kinematic chains has to take this actual dependence into account. This can be achieved with the following two steps:

(i) The IK for the virtual RCM-based chain are solved to reach the desired end-effector pose. This step provides the joint-values of post-RCM-chain and furthermore the intermediate pose ${\varvec{T}}_{{\text {vir}},{\text {p}}}$ after the 4DOF virtual base.
(ii) The IK for the pre-RCM-chain are solved to reach this intermediate pose ${\varvec{T}}_{{\text {vir}},{\text {p}}},$ which provides the joint-values of the pre-RCM-chain such that all joint-values of the complete kinematic chain are determined.

The initial joint-values of the 4DOF virtual base can be determined with an algebraic inverse-kinematic approach based on the equality:

1 $${\user2{T}}_{{{\text{vir}},{\text{p}}}} \mathop = \limits^{!} {\user2{T}}_{{{\text{pre}},{\text{e}}}}.$$

The base of the virtual RCM-based manipulator is calculated as:

2 $${\varvec{T}}_{{\text {vir}},{\text {b}}} = {\varvec{T}}_{\text {rcm}}\, {\varvec{T}}_{{\text {vir}},{\text {init}}},$$

where ${\varvec{T}}_{\text {rcm}}$ is the transformation matrix specifying the RCM-frame, and ${\varvec{T}}_{{\text {vir}},{\text {init}}}$ the initial matrix which is necessary to orient the base of the virtual RCM-based manipulator correctly for the ball-joint DH-table. The two steps can be computed as follows:

3 $${\dot{\varvec{x}}}_{{\text {vir}},{\text {e}}}= {} \Updelta _{x}\left( {\varvec{T}}_{{\text {d}},{\text {e}}} ,\, {\varvec{T}}_{{\text {vir}},{\text {e}}} \right) ,$$

4 $${\dot{\varvec{q}}}_{\text {vir}}= {} {\varvec{J}}_{\text {vir}}^{\dagger }\, {\dot{\varvec{x}}}_{{\text {vir}},{\text {e}}},$$

5 $${\varvec{q}}_{\text {vir}}(t+1)= {} {\varvec{q}}_{\text {vir}}(t) + \Updelta t\,{\dot{\varvec{q}}}_{\text {vir}},$$

6 $${\varvec{T}}_{{\text {vir}},{\text {p}}}= {} f_{{\text {vir}},{\text {p}}}\left( {\varvec{q}}_{\text {vir}}(t+1)\right) ,$$

7 $${\dot{\varvec{x}}}_{{\text {pre}},{\text {e}}}= {} \Updelta _{x}\left( {\varvec{T}}_{{\text {vir}},{\text {p}}} ,\, {\varvec{T}}_{{\text {pre}},{\text {e}}} \right) ,$$

where ${\varvec{T}}_{{\text {d}},{\text {e}}}$ is the desired user-defined end-effector pose, $\Updelta _{x}$ is a function which provides the 6DOF task-space velocity necessary to transition from the second to the first homogeneous transformation matrix, and $f_{{\mathcal {C}},i}$ is providing the *i*th intermediate transformation matrix from the forward kinematics of chain ${\mathcal {C}}.$ The pseudo-inverse of the Jacobian matrix is denoted as ${\varvec{J}}^{\dagger }.$ The joint-values for the complete kinematic chain are obtained by a combination of the pre-RCM-chain and post-RCM-chain,

8 $${\user2{q}}\mathop = \limits^{{(9)}} \left[ {\begin{array}{cc} {{\user2{q}}_{{{\text{pre}}}}^{{\text{T}}} } &\quad {{\user2{q}}_{{{\text{pos}}}}^{{\text{T}}} } \\ \end{array} } \right]^{{\text{T}}},$$

where the post-RCM-chain joint-values are a subset of the virtual RCM-based chain joint-values:

9 $${\varvec{q}}_{\text {vir}} = \left[ {\varvec{q}}_{{\text {vir}},{\text {b}}}^{\text {T}} {\varvec{q}}_{\text {pos}}^{\text {T}}\right] ^{\text {T}}.$$

Using this method, the RCM constraint is respected because the virtual base ensures that the instrument shaft is always passing through the RCM and at the same time the end-effector pose is optimized to reach the desired pose.

The method further allows the virtual RCM-based chain to be restricted to less than 4DOF. In the experiments presented in this paper only the prismatic joint was used, since the approach angle into the oesophagus should not change. The virtual RCM-based tool therefore has 8DOF consisting of the virtual 1DOF prismatic base, and the 7DOF $i^{2}Snake$ robot. The pre-RCM-chain in this experiment is actuated using the 7DOF KUKA robot. Finally, the mapping between the master interface motion and the robot tip motion is done using a ‘joint limit based Jacobian modification’ approach.^2^ This approach allows controlling the $i^{2}Snake$’s body in an intuitive way where the hand motions are replicated by the robot’s tip.^2^

#### Global Positioning

The combined robotic platform can be moved with little effort through a hands-on manipulation scheme. Using the force/torque sensing system described previously, the forces and torques applied on the handle are detected and used to move the robot arm. Generating motion based on the forces and torques detected is an admittance control problem that is solved as described below.

The system’s Cartesian position ${\varvec{x}}(t)$ at a time *t* is modeled following Newton’s second law, as a solid of mass *m* under the influence of a viscous friction with coefficient *f*,  and an effective external force ${\varvec{F}}_{\mathbf{eff}}(t).$ As the system should feel weightless, gravitational effects are considered null:

10 $${\varvec{F}}_{\mathbf{eff}}(t) = m\cdot \frac{d^{2}{\varvec{x}}}{dt^{2}}(t) + f\cdot \frac{d{\varvec{x}}}{dt}(t).$$

The transfer function of the system can be obtained by using the Laplace transform on Eq. 10:

11 $$\frac{{\varvec{X}}(s)}{{\varvec{F}}_\mathbf{eff}(s)} = \frac{1}{m\cdot s^{2} + f\cdot s}.$$

Note that in this representation ${\varvec{F}}_\mathbf{eff}(t)$ includes static friction effects such as Coulomb friction. Let ${\varvec{F}}_\mathbf{ext}(t)$ be the external force applied on the system, $f_{\text {c}}$ be the Coulomb friction coefficient, and ${\varvec{v}}(t) = \frac{d{\varvec{x}}}{dt}(t).$ The resulting Coulomb friction force ${\varvec{F}}_\mathbf{C}(t)$ can take three different expressions:

12 $$\begin{aligned} {\varvec{F}}_\mathbf{C}(t)= \left\{ \begin{array}{ll} {\varvec{F}}_\mathbf{ext}(t) &{}\quad {\text{ if } } ||{\varvec{F}}_\mathbf{ext}(t) ||< f_{\text {c}} \text{ and } ||{\varvec{v}}(t) ||< \varepsilon _{\text {v}}, \\ f_{\text {c}}\cdot \frac{{\varvec{F}}_\mathbf{ext}(t)}{||{\varvec{F}}_\mathbf{ext}(t) ||} &{}\quad \text{ if } ||{\varvec{F}}_\mathbf{ext}(t) ||\ge f_{\text {c}} \text{ and } ||{\varvec{v}}(t) ||< \varepsilon _{\text {v}}, \\ f_{\text {c}}\cdot \frac{{\varvec{v}}(t)}{||{\varvec{v}}(t) ||} &{}\quad \text{ otherwise }, \end{array} \right. \end{aligned}$$

where $\varepsilon _{\text {v}}$ is a small constant for numerical stability. The continuous-time transfer functions are discretized into Z-transform representations using the bilinear transformation. These discrete-time expressions were implemented in C++ with a discrete time-step of 50 $\mu$s. The above description only treats the case of motion generated from forces applied on the system. However, the approach presented can be extended to treat the case of torques by using the rotational equivalent of (10) for point particles: ${\varvec{\tau }} = I {\varvec{\alpha }},$ where ${\varvec{\tau }}$ are the torques applied on a rigid body, *I* is the body’s moment of inertia, and ${\varvec{\alpha }}$ is its angular acceleration. A Coulomb friction torque model can also be used to model the corresponding friction effects.

#### Cluttered Environment Compensation/Null-Space Control

As mentioned previously, the redundancy of the robotic arm can be exploited to improve the usability of the system in the operating room, through the use of a null-space control. The joint values of the robotic arm are determined using a damped least squares inverse kinematic solver, where the end-effector position and orientation are dictated by the virtual RCM as presented in “Combined *i*^2^*Snake*-KUKA Control” section. Let ${\dot{\varvec{q}}}$ denote the joint velocities of the robotic arm and ${\varvec{v}}_{\text {e}}$ the task space velocity of the robot end-effector. A Jacobian matrix ${\varvec{J}}$ can be defined that relates ${\dot{\varvec{q}}}$ and ${\varvec{v}}_{\text {e}}$:

13 $$\begin{aligned} {\varvec{v}}_{\text {e}} = \left[ \begin{array}{c} {\dot{{\varvec{p}}}}_{\text {e}} \\ {\varvec{w}}_{\text {e}} \end{array}\right] = {\varvec{J}}({\varvec{q}}){\dot{\varvec{q}}}, \end{aligned}$$

where ${\varvec{q}}$ are the robot arm joint values, ${\dot{\varvec{p}}}_{\text {e}}$ is the linear velocity of the robot arm end effector, and ${\varvec{w}}_{\text {e}}$ is its angular velocity.^22^ The inversion of the matrix ${\varvec{J}}$ can be ill-defined depending on the robot configuration, such as when nearing kinematic singularities or trying to reach points outside the workspace. As such, a damped pseudo-inverse ${\varvec{J}}^{*}$ of the Jacobian matrix ${\varvec{J}}$ is used to to avoid high joint velocities near singularities.^10^,^15^ The joint velocities can then be expressed as:

14 $${\dot{\varvec{q}}} = {\varvec{J}}^{*}{\varvec{v}}_{\text {e}}.$$

Using the notation above, the matrix $({\varvec{I}} - {\varvec{J}}^{*}{\varvec{J}})$ is a null-space projector, i.e., it is a matrix which projects an arbitrary vector ${\dot{\varvec{q}}}_{0}$ in the null space of ${\varvec{J}}.$ As null space motions do not affect the end-effector position and orientation, ${\dot{\varvec{q}}}_{0}$ effectively acts as a constraint on ${\dot{\varvec{q}}}$ that the system will try to respect by using the redundancy of the robot. Equation (14) can be reformulated to take advantage of this redundant behavior:

15 $${\dot{\varvec{q}}} = {\varvec{J}}^{*}{\varvec{v}}_{\text {e}} + ({\varvec{I}} - {\varvec{J}}^{*}{\varvec{J}}){\dot{\varvec{q}}}_{0}.$$

In the presented system the force detected on the handle is used to generate a desired velocity ${\dot{q}}_{3}$ for the third joint of the robot arm:

16 $${\dot{q}}_{3} = k {\varvec{T}}_{\mathbf{fm}} {\varvec{F}}_{\mathbf{ext}},$$

where ${\varvec{T}}_{\mathbf{fm}}$ is a $3\times 1$ transformation matrix representing the spatial mapping between the force and the third joint velocity, and *k* is a coefficient regulating the speed generated from the force.

## Results

### Teleoperation

**Figure 5:**
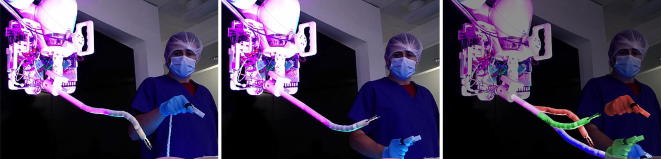
Time-lapse of the teleoperation of the platform. While the system is in the ‘teleoperation mode’, the surgeon can intuitively control the position, orientation and insertion of the $i^{2}Snake$ robot by using the master interface. The sub-figure on the right shows stacked master–slave position pairs depicted in matching colors.

The teleoperation mapping between the master and the slave was validated in simulation before being implemented on the system. The results of the teleoperation implementation are shown in Fig. 5. A change in the master’s position will induce a corresponding change of the robotic platform combining the motion of the KUKA with the motion of the $i^{2}Snake.$ The position and orientation motion are decoupled from each other, so a change in position will not affect the orientation of the head of the $i^{2}Snake$ and *vice versa*. This approach is standard in master–slave systems and allows a more intuitive navigation while looking at the camera image, as hand motions correspond to the camera motion seen on the monitor. It also allows the snake robot to take S-shapes by moving upward while tilting downwards for instance, as shown in the left-hand sub-figure of Fig. 5. The insertion into the mouth cavity is simply performed by moving the hand forward and horizontally toward the monitor.

#### Inverse Kinematic

**Figure 6:**
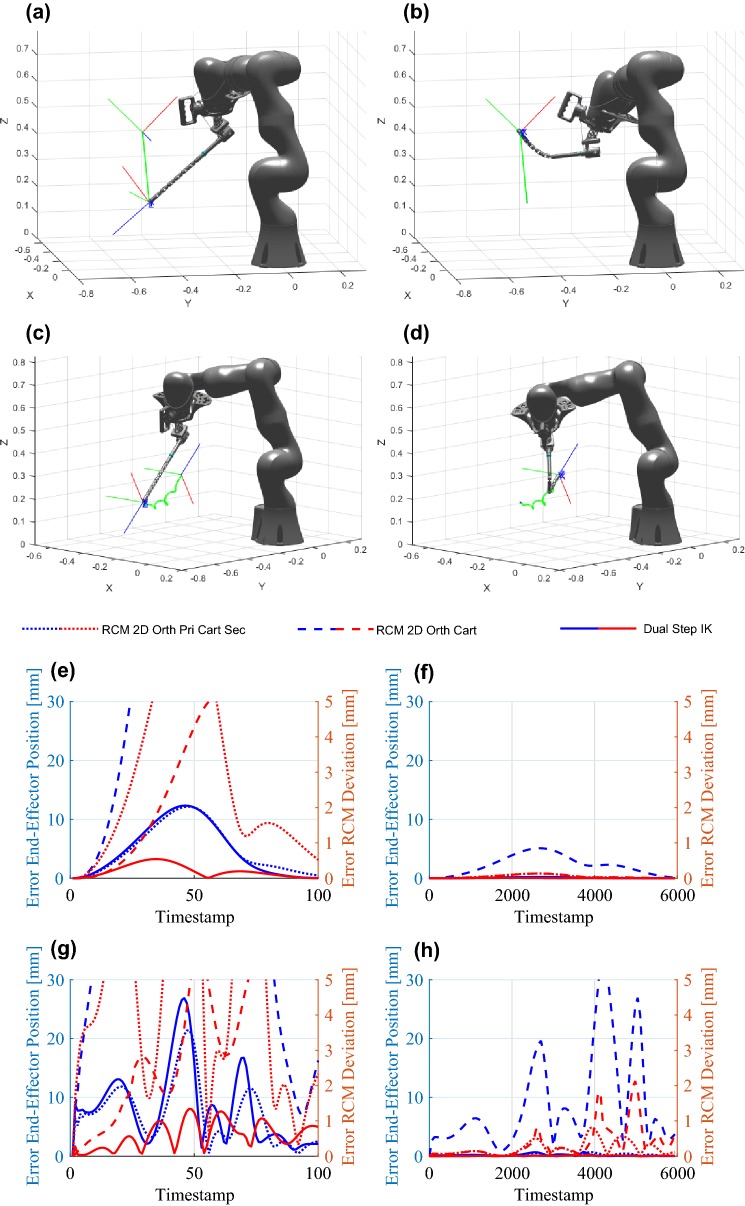
Trajectories used in the inverse kinematic evaluation simulation. Line trajectory from (a), (b). Screw trajectory from (c), (d). The cyan sphere represents the RCM. (e)–(h) Error of the inverse kinematic techniques. (e), (f) Linear trajectory with 100 and 6000 time samples respectively. (g), (h) Screw trajectory with 100 and 6000 time samples, respectively.

The inverse kinematic approach described was further evaluated through simulation. Two evaluated scenarios are depicted in Fig. 6. One trajectory represents a motion with a normal insertion vector from Figs. 6a and 6b. The second trajectory represents a screw motion while retroflexing from Figs. 6c and 6d. It was assessed how close the IK solvers would find a solution to the desired position and how far the shaft deviated from the incision point. Furthermore, the trajectory was divided into 100 and 6000 samples/time steps, which represents a constant velocity trajectory. The inverse kinematic approach presented here (*Dual Step IK*) was compared to the approach presented by Locke and Patel^12^ where the Cartesian task-space is extended by 2DOF orthogonal to the incision normal (*RCM 2D Orth Cart*) to an overall 8DOF task-space, and further compared to the approach of Azimian *et al.*^1^ where the 2DOF task-space orthogonal to the incision normal represents the primary goals and the 6DOF task-space Cartesian task-space secondary goals (*RCM 2D Orth Pri Cart Sec*). The simulation results are presented Figs. 6e–6h. In Figs. 6e and 6f the results for the line trajectory are presented with 100 and 6000 samples, respectively and in Figs. 6g and 6h the results for the screw trajectory. All algorithms show better results when more time samples are used, which is expected, as the step per IK iteration is significantly smaller with more samples. Furthermore, it can be seen that in these scenarios the *Dual Step IK* approach outperforms the other two approaches. The *RCM 2D Orth Pri Cart Sec* approaches suffers mainly from its slow convergence due to optimizing for Cartesian goals in the null-space of the RCM constraint goals.

### Global Positioning

**Figure 7:**
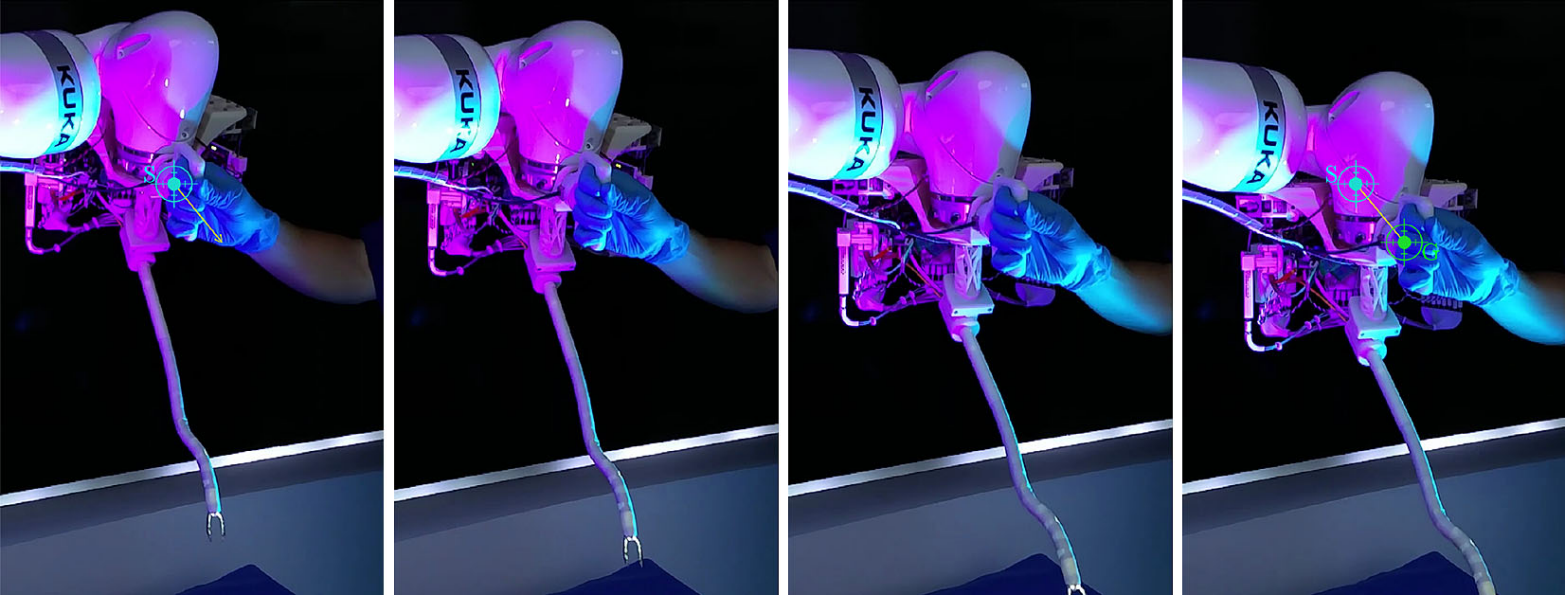
Global positioning mode time lapse. While the system is in the global positioning mode, the surgeon can intuitively control the position and orientation of the platform by using the handle equipped with the force sensor. The platform will move along the force vector from the start point S to the goal point G.

The results of the hands-on control mode are shown in the time-lapse (Fig. 7). It can be seen that the KUKA robot’s position and orientation can be intuitively controlled by holding the handle equipped with the force sensor (while pressing on the desired foot pedal) and by manually guiding the system toward a goal position. The direction of the force applied on the handle will determine the motion direction of the robot’s end effector, while the torques applied on the handle will induce a corresponding change in its orientation. Once the system in position, releasing the handle or the pedal will leave the robot in place, ready for the teleoperated insertion phase.

**Figure 8:**
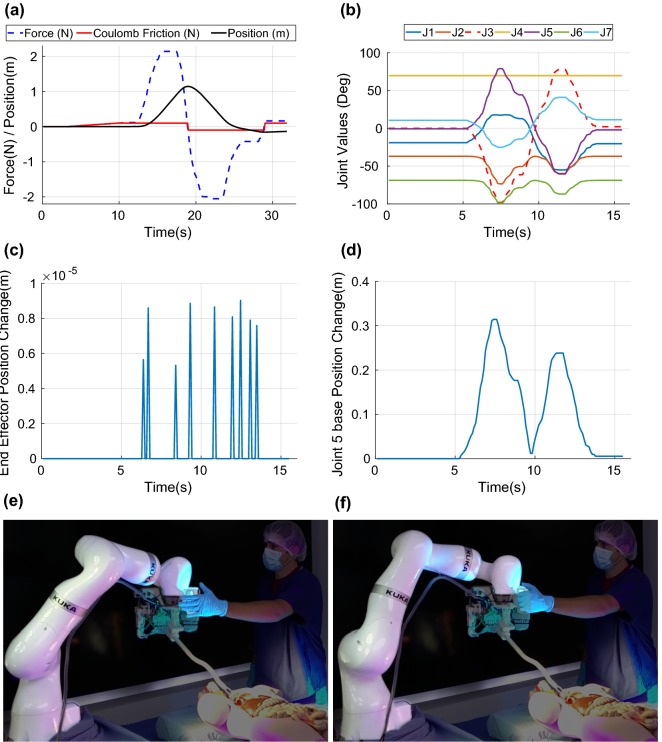
Characterization of the force-based global positioning and the null-space control. (a) Example 1-dimensional trajectory generated by a force exerted on a single axis of the handle, (b) joint values during an example null-space motion, (c) distance of the end-effector from its starting position during the null-space motion generated in (b), (d) distance of the ‘elbow’ of the KUKA robot (base of joint 5) from its starting position during the null-space motion generated in (b), (e)– (f) example of the motion generated by the null-space control, moving the ‘elbow’ of the robot through forces applied on the handle while keeping the end-effector stationary.

An example of the behavior of the global positioning control is shown in Fig. 8a. This figure illustrates how the Coulomb friction prevents low forces from generating motions, and how smooth trajectories are generated by the inductance controller. The following values were used here: mass $m=0.5$ kg, viscous friction $f=8.0\,{\text {kg}}\,{\text {s}}^{-1},$ Coulomb friction coefficient $f_{\text {c}}=0.1$ and $\varepsilon _{\text {v}} = 10^{-9}.$

### Null-Space Control

The null-space control was implemented in simulation before being tested on the system. The results are shown in Figs. 8e and 8f. By using the information of the force sensor, combined with the foot pedal, the surgeon can intuitively move the redundant joints of the KUKA robot without moving the snake robot. The direction of the force, as shown by the finger location in Figs. 8e and 8f, will move the third joint of the KUKA robot in the corresponding direction, allowing to clear the space occupied by the body of the KUKA robot and limit the potential risk of collision with other equipment.

Figures 8b–8d illustrate the behavior of the null-space control. In Fig. 8b, the third joint is controlled as detailed in “Cluttered Environment Compensation/Null-space Control” section to follow a trajectory generated based on the forces applied to the handle. The other joints move accordingly, allowing the end-effector to remain steady. Note that the fourth joint remains at the same value during the entire null-space motion. This is due to the fact that, for this particular robot configuration, the fourth row and column of the null-space mapping matrix ${\varvec{I}} - {\varvec{J}}^{*}{\varvec{J}}$ are composed of zeroes or negligible values. As a result, there is no ${\dot{\varvec{q}}}_{0}$ that can generate a motion on the fourth joint, and secondary goals depending on the fourth component of ${\dot{\varvec{q}}}_{0}$ cannot be fulfilled. This effectively means that, for this robot configuration, motions on the fourth joint cannot be compensated by the other joints and will result in a movement of the end-effector. Figure 8c shows that there is no motion of the end-effector greater than 10 $\mu$m, which is sufficient for ENT surgery. These punctual errors are likely due to inaccuracies in the kinematics solver and could be further reduced by increasing the number of optimization iterations in the solver until they fall under a specific threshold. Lastly, Fig. 8d illustrates the distance traveled by the ‘elbow’ of the KUKA robot during this null-space motion. This distance is of course entirely dependent on the joint configuration, but serves to indicate that the robot body can easily be shifted by tens of centimeters while keeping the end-effector steady.

## Discussion

This paper presents the Intuitive Imaging Sensing Navigated and Kinematically Enhanced Robotic Platform for ENT Surgery: The $i^{2}Snake.$ This robotic platform aims at providing the surgeon with an enhanced robotic endoscope allowing to perform more complex endoscopic procedures. The proposed snake robot system has four channels that can be used for robotic instruments, camera and light source, and suction/irrigation. The presented work focused mainly on the design, the ergonomics and the clinical application. The robotic platform was designed to be user-friendly and portable. The snake robot weights less than 4 kg, and if positioned on a robotic arm, the whole system can easily be maneuvered with the use of a wheeled cart. The master interface is also lightweight and offers a large convenient workspace, reducing the need for clutching. Although the system is fully functional, further work needs to be carried on all the components before it can be used in clinical trial. Some of the limitations and future work are described in the following sections.

### The $i^{2}Snake$

The $i^{2}Snake$ being at an early stage of development, there are some challenges left to overcome before the system can be used in clinical applications. As the $i^{2}Snake$ is tendon driven, it is subject to backlash and tendon elongation leading to positioning error. Similar types of problem have been addressed with either software^10^ or hardware solutions^7^ with significant error reduction. Future work will investigate combined software and hardware solutions and sensing^18^ for closed-loop control to compensate for this behavior and enhance the operation of the robot. The teleoperation of the redundant robot is also one of the challenges left to overcome,^2^ but hybrid approached combining motion planners and navigation algorithms^9^ could improve the control and will be investigated. Ultimately, advances made in the field of snake-like robots control would lead to the possibility to perform autonomous safe navigation toward the surgical site of interest.

### Robotic Arm

The use of a robotic arm to hold an endoscopic robot has already been investigated previously.^19^ Other systems either use a traditional endoscopic approach where the endoscope is manually inserted^13^,^27^ or a custom platform.^4^ The robot holder could also be as simple as a linear stage allowing to insert the snake inside the mouth cavity. The advantage of using a redundant robotic arm is to enhance the snake robot with features such as dynamic motion compensation.^8^ To cater for patient or physiological motion during the surgery, the KUKA robot could be used to compensate for that motion resulting in a better safety and more stability of the end effector during the surgery. In future applications, the robotic arm could also be used to find the optimal $i^{2}Snake$ position to perform a pre-planned surgery and maximize the available dexterity. This would require knowing the patient’s position and orientation as well as the relative position of the surgical site. However, such approach would result in the robot moving autonomously, which in practice would make obtaining regulatory approval more challenging, whereas manual positioning by the surgeon is a common practice for any medical device.

### Robotic Instruments

The robotic instruments are at an early stage of development and although they are functional, further work needs to be done on characterization and control. Cross talk between the snake’s body and the instruments and within the instrument itself is a known problem. This behavior affects the path length of the tendons leading to undesired motions of the instrument’s tip. Future work will focus on addressing these issues by using compensation techniques^7^,^10^,^18^ for a better accuracy and more reliable instruments. Future work will provide the instruments with an additional axial rotation and translation to further increase the dexterity.

### Master Interface and Hands-on Control

The use of a handle positioned on the robotic arm for global positioning is a simple yet intuitive way to control the system. This approach was proposed on a similar type of manipulator^24^ and appears to be more straightforward to use that using a separate master interface.^19^ Regarding the presented master interface, as it uses EM signals, it is sensitive to ferromagnetic metals in the surroundings which will create distortions and impact the overall accuracy of the system.^14^ Moreover, the master interface requires wires to transmit the signal to the control board. Future work will investigate wireless options with similar key features such as large workspace, hand-held and user-friendly design, but that will use other means of sensor tracking such as inertial motion units combined with visual tracking.

## Abbreviations

DOF: Degree of freedom
EM: Electromagnetic
ENT: Ear–nose–throat
ESD: Endoscopic submucosal dissection
$i^{2}Snake$: Intuitive imaging sensing navigated and kinematically enhanced
IK: Inverse kinematics
MIS: Minimally invasive surgery
POEM: Peroral endoscopic myotomy
RCM: Remote centre of motion
